# Efficacy and safety of anterior transposition of the ulnar nerve for distal humerus fractures: A systematic review and meta-analysis

**DOI:** 10.3389/fsurg.2022.1005200

**Published:** 2023-01-06

**Authors:** Ting Li, Jingxin Yan, Qiuyu Ren, Jiang Hu, Fei Wang, Chengwei Xiao, Xilin Liu

**Affiliations:** ^1^ Department of Orthopedics, Sichuan Provincial People's Hospital, Chengdu, China; ^2^ Department of Postgraduate, Chengdu Medical College, Chengdu, China; ^3^ Department of Interventional Therapy, Affiliated Hospital of Qinghai University, Xining, China; ^4^ Department of Postgraduate, Qinghai University, Xining, China

**Keywords:** meta-analysis, anterior transposition, ulnar nerve (MeSH), distal humerus fractures, effect

## Abstract

**Background:**

This systematic review and meta-analysis was performed to summarize available evidence of anterior transposition of the ulnar nerve for patients with distal humerus fractures.

**Materials and Methods:**

The databases were searched from PubMed, Cochrane, Embase, Scopus, Web of Science, Chinese National Knowledge Infrastructure (CNKI), Chongqing VIP Database (VIP), and Wan Fang Database up to June 2022. The clinical outcome included operation time, fracture healing time, hospital stays, elbow joint function, and ulnar neuritis rate. Statistical analysis was performed with Review Manager 5.3 (Cochrane Collaboration).

**Results:**

A total of 17 studies were included (8 RCTs and 9 retrospective studies), and 1280 patients were analyzed. The results of this meta-analysis showed anterior transposition group had longer operation time (MD = 20.35 min, 95%CI: 12.56–28.14, *P* < 0.00001). There was no significant difference in fracture healing time (SMD = −0.50, 95%CI: −1.50–0.50, *P* = 0.33), hospital stays (MD = −1.23 days, 95%CI: −2.72–−0.27, *P* = 0.11), blood loss (MD = 2.66 ml, 95%CI: −2.45–7.76, *P* = 0.31), and ulnar neuritis rate (OR = 1.23, 95%CI: 0.63–2.42, *P* = 0.54) between two groups. Finally, elbow joint motion, elbow joint function, fracture nonunion, and post-operative infection (*P* > 0.05) between two groups were not significantly statistic difference.

**Conclusion:**

This meta-analysis showed that anterior transposition group is not superior to non-transposition group for patients with distal humerus fractures without ulnar nerve injury. On the contrary, non-transposition group have shorter operation time than that of anterior transposition group. Non-transposition group did not increase the post-operative ulnar neuritis rate. Therefore, both anterior transposition group and non- transposition group are the treatment options for patients with distal humerus fractures without ulnar nerve injury. Besides, these findings need to be further verified by multi-center, double-blind, and large sample RCTs.

## 1. Introduction

Elbow joint fractures account for approximately 7% of adult fractures, with distal humerus fractures accounting for 30% of all elbow joint fractures (1). When open reduction and internal fixation (ORIF) is performed, several complications such as fracture nonunion, loss of functional motion, and ulnar neuropathy have been reported (2, 3). Ulnar neuropathy in particular poses a unique challenge. The incidence of ulnar neuropathy following ORIF of distal humerus fractures has been reported between 0%–51% in previously described studies (4). Some literatures have reported that when the patients with distal humerus fracture complicated with ulnar nerve injury, anterior transposition of the ulnar nerve was more effective in promoting postoperative nerve rehabilitation (5, 6). Duo to the ideal clinical effect of anterior transposition of the ulnar nerve, most orthopedic surgeons have taken an anterior transposition of the ulnar nerve as a treatment for distal humeral fractures to reduce the possibility of postoperative ulnar nerve disorders (7).

However, in recent years, there has been some different views on whether the anterior transposition of the ulnar nerve is necessary in patients without ulnar nerve injuries. Chen et al. (8) reported a retrospective study of 137 distal humeral fractures patients who had no ulnar nerve injuries before surgery, and their results showed that the incidence of postoperative ulnar nerve disorders was 4 times higher in the anterior transposition group than in the non-transposition group. On the contrary, Ruan et al. (9) reported that according to Bishop rating system, postoperative excellent results of ulnar nerve function were achieved in patients (86.7%) in the transposition group, compared with patients (57.1%) in the decompression group (*P* < 0.05).

However, it is still controversial whether the anterior transposition of the ulnar nerve is necessary for patients without ulnar nerve injuries before surgery. We aim to conduct a systematic review and meta-analysis including current literature to assess the benefits and risks by comparing anterior transposition of the ulnar nerve with non-transposition treatment for patients with distal humerus fractures.

## 2. Material and methods

### 2.1. Study design

This meta-analysis was by the PRISMA statement (10).

### 2.2. Search strategy

The following relational databases were searched up to June 2022 such as PubMed, Cochrane, Embase, Scopus, Web of Science, CNKI, VIP, and Wan Fang. All RCTs and retrospective studies comparing anterior transposition of the ulnar nerve and non-transposition for the treatment of distal humerus fractures were collected. The retrieval method adopted the combination of subject words and free words, and English retrieval words and Chinese versions include: (((Anterior ulnar nerve [Title/Abstract]) OR (Ulnar neuritis [Title/Abstract])) OR (Ulnar nerve [Mesh])) AND ((((humerus fracture [Mesh]) OR (Humeral supracondylar fracture [Title/Abstract])) OR (Humeral intercondylar fracture [Title/Abstract])) OR (Distal humerus fractures [Title/Abstract])). In addition, the references of the included literature were reviewed to supplement the relevant studies.

### 2.3. Inclusion and exclusion criteria

#### 2.3.1. Inclusion criteria

Studies were eligible for inclusion if they met the following criteria: (1) P: all patients were diagnosed by clinical and radiographic confirmation of distal humerus fractures without ulnar nerve injury. (2) I: interventions group used a plate fixation plus anterior transposition of the ulnar nerve for patients with distal humerus fractures. (3) C: control method used a plate fixation plus non-transposition of the ulnar nerve for patients with distal humerus fractures. (4) O: availability of adequate raw data for categorical outcomes. (5) S: study design included RCTs and retrospective studies.

#### 2.3.2. Exclusion criteria

Studies were ineligible if they met the following criteria: (1) studies cannot extract data studies so that the study could not be analyzed; (2) duplicate studies; (3) other interventions or no-operation treatment; (4) relevant clinical outcome were not reported; (5) animal studies, duplicate publications of one trial, case report, case series, letter, technology note, commentaries, reviews, withdraw trails and meta-analysis.

### 2.4. Data extraction

Two researchers independently read potential studies based on the inclusion and exclusion criteria. The data were extracted as follows: the basic information of the study sample (name, year, gender, the number of participants, intervention method, control method, etc.), study design type (RCTs and NRCTs), study duration, AO fracture classification, Surgery approach etc. What's more, we extracted the following data from each selected study: operation time, blood loss, hospital stays, elbow joint function, elbow joint motion, fracture healing time, and ulnar neuritis rate. When information was missing, we attempted to contact the primary author *via* email to seek clarification or exclude the study.

### 2.5. Risk of bias assessment

Using the Cochrane risk of bias tool to evaluated the risk of bias in the included studies for RCTs. And using Newcastle-Ottawa Scale to evaluated retrospective study. The studies with scored ≥ 6 were considered to be high-quality articles for retrospective studies. The bias assessment was conducted by two independent researchers. Any unresolved disagreements between reviewers were resolved through discussion or assessment by a third reviewer.

### 2.6. Statistical analysis

The Revman 5.4 software package was used. We reported continuous outcomes for mean difference (MD) or standardized mean difference (SMD) with 95% confidence interval (CI), and the dichotomous outcomes were reported by odds ratio (odds ratio, OR) with 95% CI. Chi-square test was used to test the heterogeneity of the included research results. There was heterogeneity between studies if *P* < 0.1 or *I*^2 ^> 50%, and a random effect model was used. If *P* ≥ 0.1 and *I*^2^ ≤ 50%, it showed that there was no heterogeneity. Therefore, a fixed-effect model was used. We also performed a sensitivity analysis to determine the resource of the heterogeneity. Publication bias was assessed by the funnel plot.

## 3. Results

### 3.1. Search result

**Figure 1:**
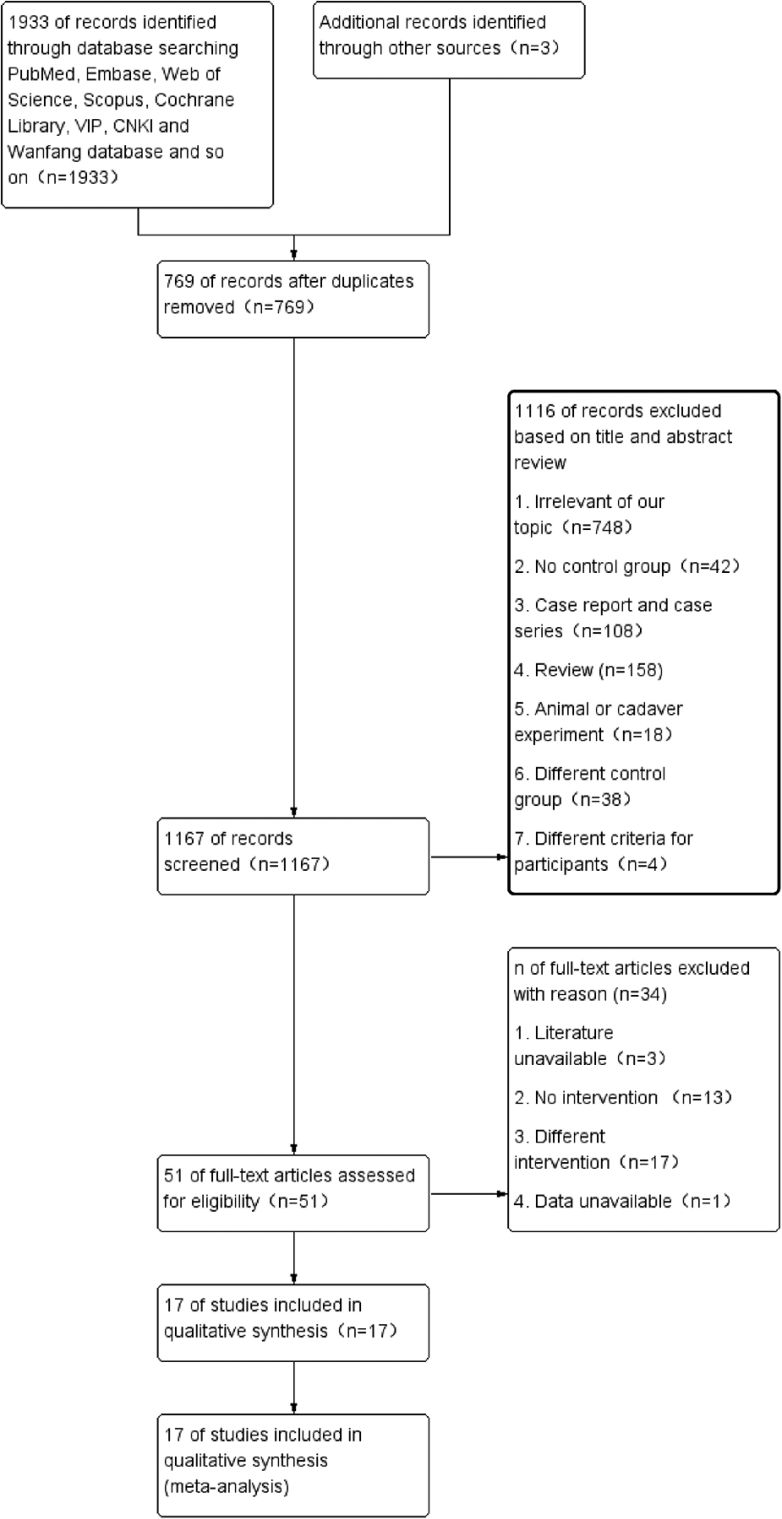
The flowchart of the study.

The initial search produced 1936 records, from which we excluded 769 records due to the duplication. After reading the full-text articles, 17 potentially eligible studies were included. Figure 1 shows the selection algorithm, numbers of included and excluded studies. All titles, abstracts, and text were dually and independently reviewed by the authors based on the inclusion and exclusion criteria to minimize bias.

### 3.2. Study characteristic

Among the included studies, 8 were RCTs (9, 11–17) and 9 were retrospective studies (7, 8, 18–24). There were 1280 patients included in this meta-analysis. After the application of the inclusion criteria, 8 trials published in English and 9 trials published in Chinese were included in this meta-analysis. The main basic characteristics of the included literature were shown in Table 1.

**Table 1:** Basic characteristics of the included literature.

Name	Year	Age (I/C)	Study type	Number of persons (I/C)	Intervention group	Controlled group	AO fracture classification	Surgery approach	Follow-up (I/C)
Ye, H	2017	35.5/43.8	RCT	36/32	Transposition ulnar nerve	No transposition ulnar nerve	A = 9; B = 7; C = 20/A = 10; B = 6; C = 16	Triceps approach/Olecranon osteotomy	12/12 Months
Bao, GD	2021	58/63	Retrospective	20/16	Transposition ulnar nerve	No transposition ulnar nerve	A = 3; B = 1; C = 16/A = 2; B = 1; C = 13	Triceps approach/Olecranon osteotomy	22/23 Moths
Meng, DQ	2015	53.5/53.5	RCT	56/57	Transposition ulnar nerve	No transposition ulnar nerve	C = 113	Olecranon osteotomy	NA
Wang, JP	2017	51.2/51.2	RCT	78/77	Transposition ulnar nerve	No transposition ulnar nerve	C = 155	Olecranon osteotomy	15/15 Months
Liu, G	2019	42.5/43.4	RCT	48/120	Transposition ulnar nerve	No transposition ulnar nerve	NA	Triceps approach/Olecranon osteotomy	32/32 Months
Cai, J	2019	40.9/41.5	Retrospective	11/12	Transposition ulnar nerve	No transposition ulnar nerve	C = 11/C = 12	Olecranon osteotomy	27/27 Months
Luo, XQ	2021	31.77/31.72	RCT	37/38	Transposition ulnar nerve	No transposition ulnar nerve	C = 37/C = 38	Olecranon osteotomy	NA
Wang, W	2017	63.8/62.1	Retrospective	43/31	Transposition ulnar nerve	No transposition ulnar nerve	A = 7; C = 36/A = 4; C = 27	Triceps approach/Olecranon osteotomy	12/12 Months
Lu, J	2011	23/22	RCT	16/16	Transposition ulnar nerve	No transposition ulnar nerve	B = 16/B = 16	Triceps approach	18/18 Months
Ahmed, AF	2020	35/36	Retrospective	28/69	Transposition ulnar nerve	No transposition ulnar nerve	A = 2; B = 4; C = 22/A = 7; B = 5; C = 57	Triceps approach/Olecranon osteotomy	11/11 Months
Barrios, C	1991	NA	Retrospective	9/6	Transposition ulnar nerve	No transposition ulnar nerve	NA	NA	NA
Chen, RC	2010	43.2/48.6	Retrospective	48/89	Transposition ulnar nerve	No transposition ulnar nerve	A = 1; C = 47/A = 7; C = 82	Triceps approach/Olecranon osteotomy	10/16 Months
Vazquez, O	2010	52/52	Retrospective	47/22	Transposition ulnar nerve	No transposition ulnar nerve	NA	NA	21/21 Months
Dehghan, N	2021	51/54	RCT	27/31	Transposition ulnar nerve	No transposition ulnar nerve	A = 4; B = 0; C = 23/A = 4; B = 1; C = 26	Triceps approach/Olecranon osteotomy	12/12 Months
Wiggers, JK	2012	57/57	Retrospective	57/50	Transposition ulnar nerve	No transposition ulnar nerve	NA	Triceps approach/Olecranon osteotomy	NA
Ruan, HJ	2009	45.1/40.7	RCT	15/14	Transposition ulnar nerve	No transposition ulnar nerve	C = 15/C = 14	Olecranon osteotomy	30/30 Months
Worden, A	2012	46/46	Retrospective	12/12	Transposition ulnar nerve	No transposition ulnar nerve	A = 7; B = 2; C = 15	Triceps approach/Olecranon osteotomy	NA

NA, not available; RCT, randomized control trial.

### 3.3. The bias risk assessment results of the included studies

**Figure 2:**
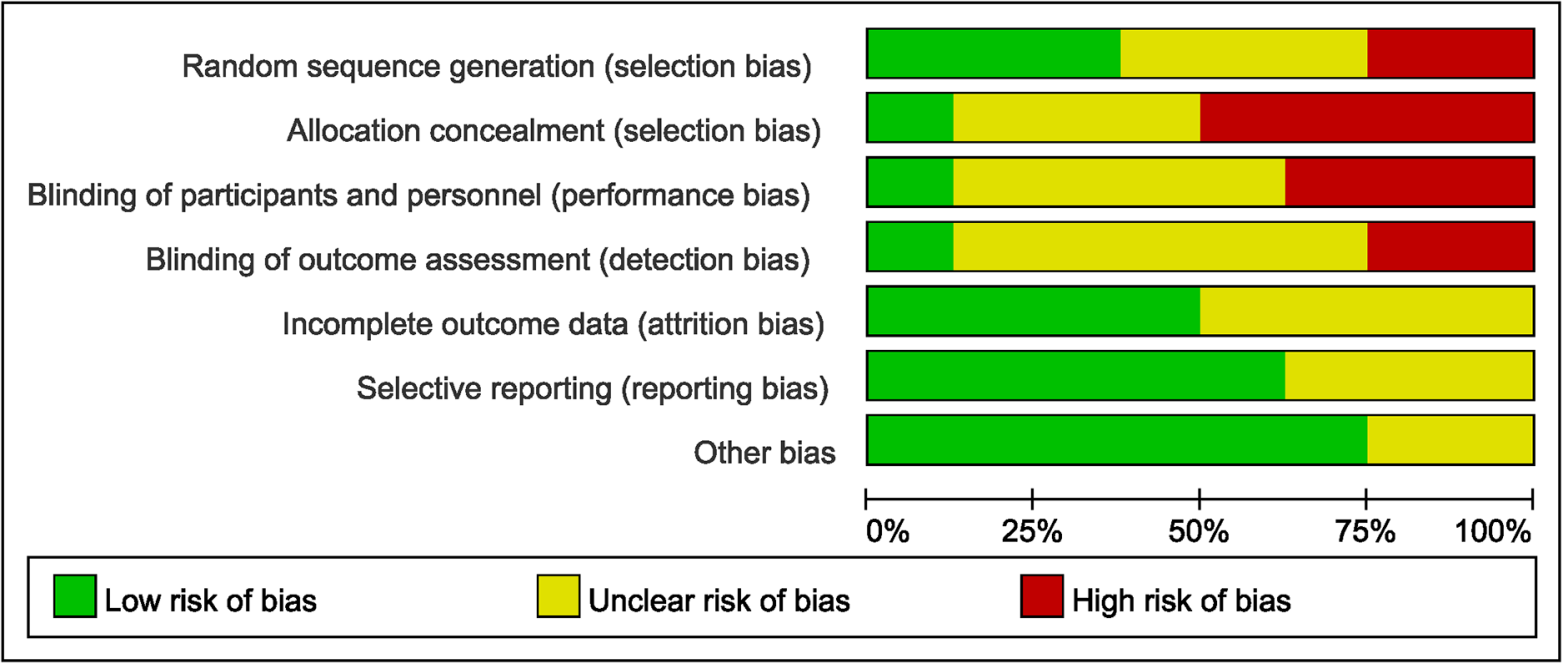
Results of quality assessment using cochrane risk of bias tool for RCTs.

In the retrospective studies, NOS scale was used to evaluate the risk of bias. The included retrospective studies met most of the quality assessment criteria, and the total scores of all studies were ≥ 6, which indicated a low risk of bias. The detail of the information could be seen in Table 2. RCTs were evaluated by using the Cochrane risk of bias tool. The quality assessment of included studies was shown in Figure 2 for details.

**Table 2:** Results of quality assessment using Newcastle–Ottawa scale for cohort studies.

Study selection	Representativeness of the exposed cohort	Selection of the nonexposed cohort	Ascertainment of exposure	Demonstration that outcome of interest was not present at start of study	Comparability of cohorts on the basis of the design or analysis	Assessment of outcome	follow-up long enough for outcomes to occur	Adequacy of follow-up of cohorts	Quality score
Bao, GD. 2021	1	1	1	1	1	1	1	1	8
Cai, J. 2019	1	1	1	1	1	1	1	1	8
Wang, W. 2017	1	1	1	1	1	1	1	1	8
Ahmed, AF. 2020	1	1	1	1	1	1	1	1	8
Barrios, C. 1991	1	1	1	1	0	1	1	0	6
Chen, RC. 2010	1	1	1	1	1	1	1	1	8
Vazquez, O. 2010	1	1	1	1	1	1	1	0	7
Wiggers, JK. 2012	1	1	1	1	0	1	1	0	6
Worden, A. 2012	1	1	1	1	1	1	1	0	7

### 3.4. Meta-analysis results

#### 3.4.1. Operation time

**Figure 3:**
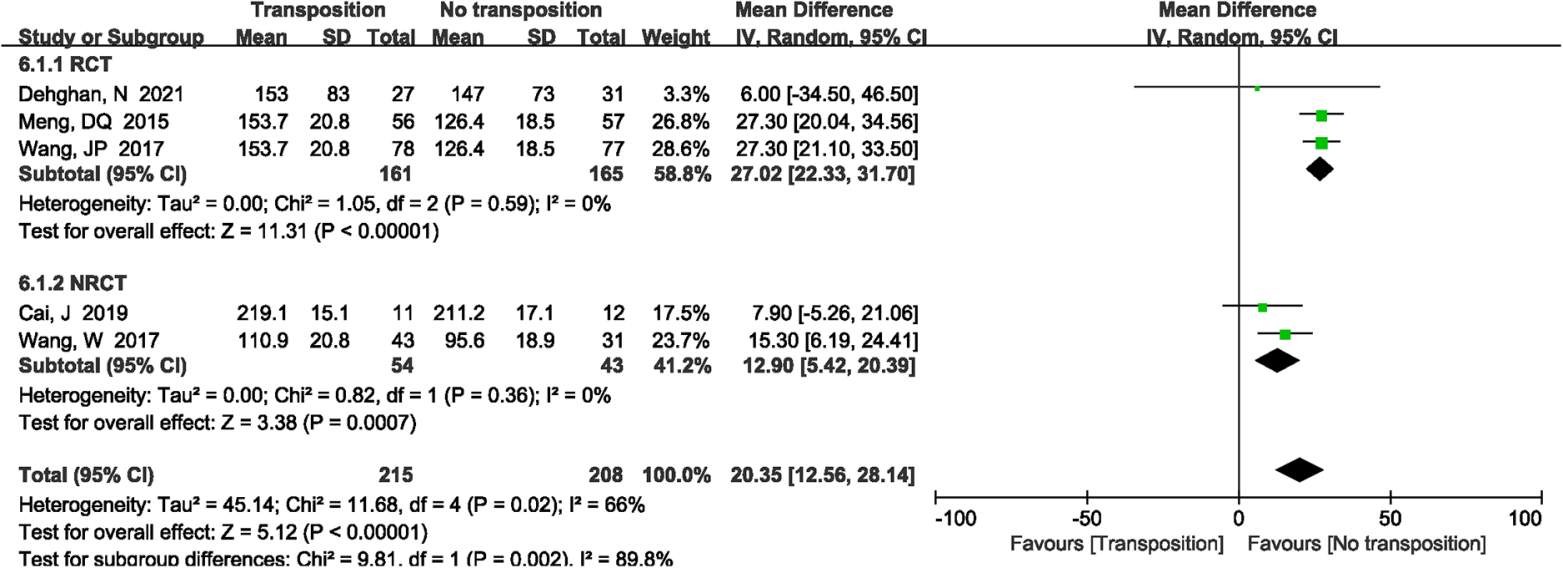
A forest plot showing the operation time.

A total of 5 studies (12, 13, 17, 19, 20) reported the operation time. There was significant heterogeneity in the study (*P *= 0.02, *I*^2^*^ ^*= 66%). Random effects model was performed. The results showed that anterior transposition group had longer operation time compared with non-transposition of the ulnar nerve (MD = 20.35 min, 95%CI: 12.56–28.14, *P* < 0.00001, Figure 3). The subgroup analysis showed that non-transposition group had lower operation time for RCTs and retrospective studies, and there was no significant heterogeneity in the study (*I*^2^*^ ^*= 0%, Figure 3).

#### 3.4.2. Blood loss

**Figure 4:**
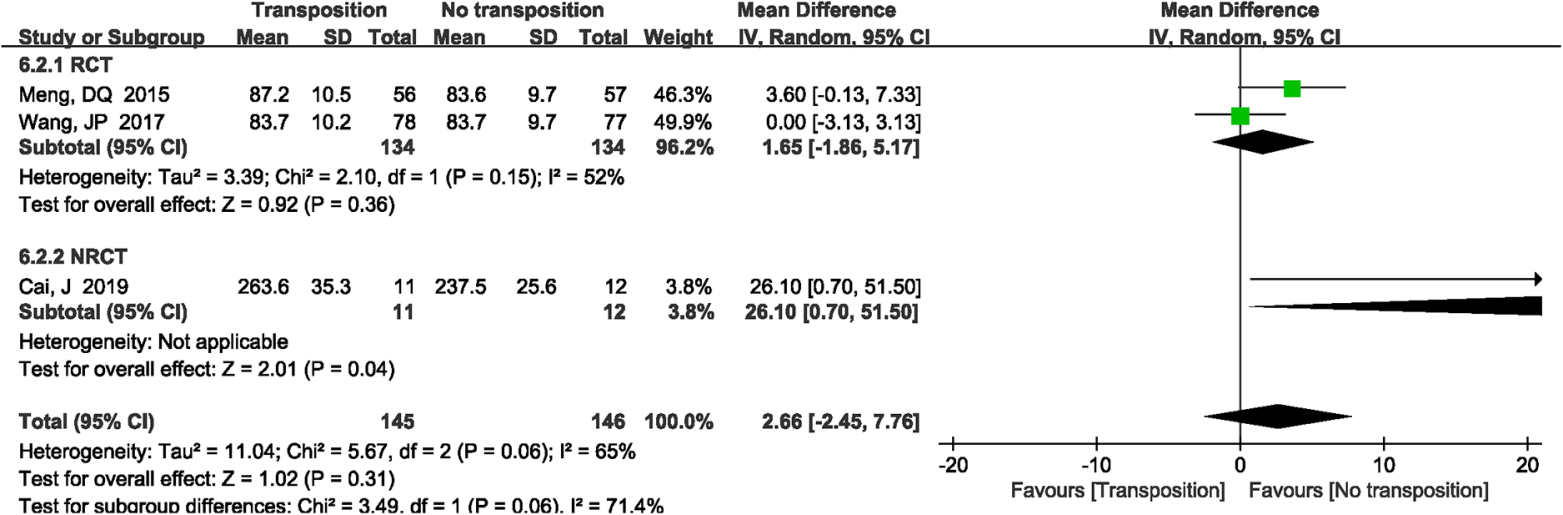
A forest plot showing the blood loss.

A total of 3 studies (12, 13, 19) reported intraoperative blood loss. There was significant heterogeneity in the study (*P *= 0.06, *I*^2^*^ ^*= 65%). Random effects model was performed. Meta-analysis results showed that there was no significant difference between anterior transposition group and non-transposition group (MD = 2.66 ml, 95%CI: −2.45–7.76, *P *= 0.31, Figure 4).

#### 3.4.3. Hospital stays

**Figure 5:**
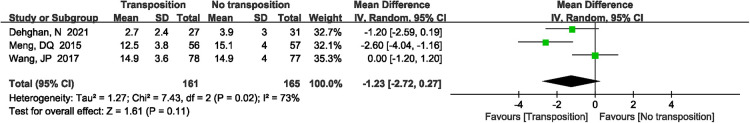
A forest plot showing the hospital stays.

A total of 3 studies (12, 13, 17) reported the hospital stays. There was significant heterogeneity in the study (*P *= 0.02, *I*^2^*^ ^*= 73%). Random effects model was performed. Results showed that anterior transposition group was not superior to non-transposition group (MD = −1.23 day, 95%CI: −2.72 ∼ 0.27, *P *= 0.11, Figure 5).

#### 3.4.4. Fracture healing time

**Figure 6:**
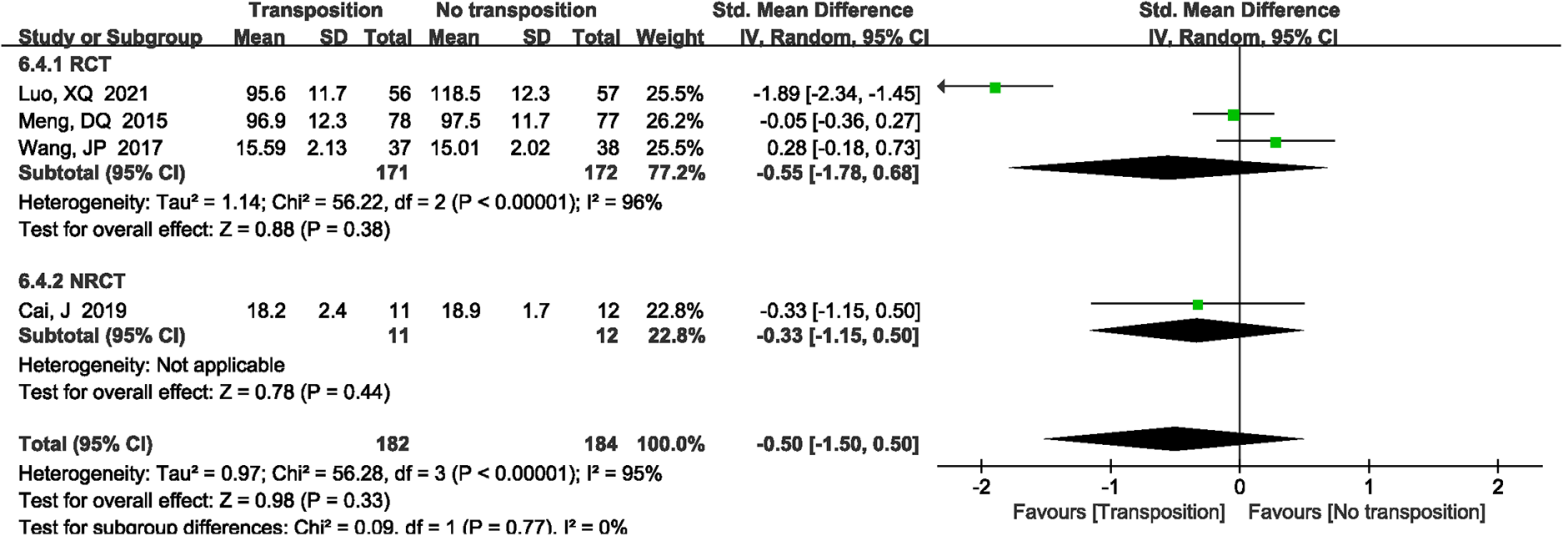
A forest plot showing the fracture healing time.

A total of 4 studies (12, 13, 15, 19) reported fracture healing time. There was significant heterogeneity in the study (*P *< 0.00001, *I*^2^*^ ^*= 95%). Random effects model was performed. There was no significant difference between two groups (SMD = −0.50, 95%CI: −1.50–0.50, *P *= 0.33, Figure 6).

#### 3.4.5. Elbow joint motion

**Figure 7:**
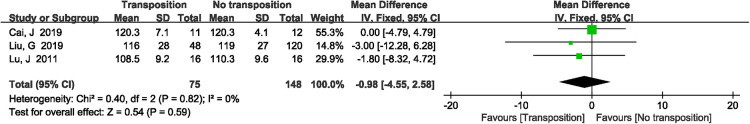
A forest plot showing the elbow joint motion.

A total of 3 studies (14, 16, 19) reported the elbow joint motion. There was no significant heterogeneity in the study (*P *= 0.82, *I*^2^*^ ^*= 0%). Fixed effects model was performed. There was no significant difference between two groups (MD = −0.98, 95%CI: −4.55–2.58, *P *= 0.59, Figure 7).

#### 3.4.6. Elbow joint function

**Figure 8:**
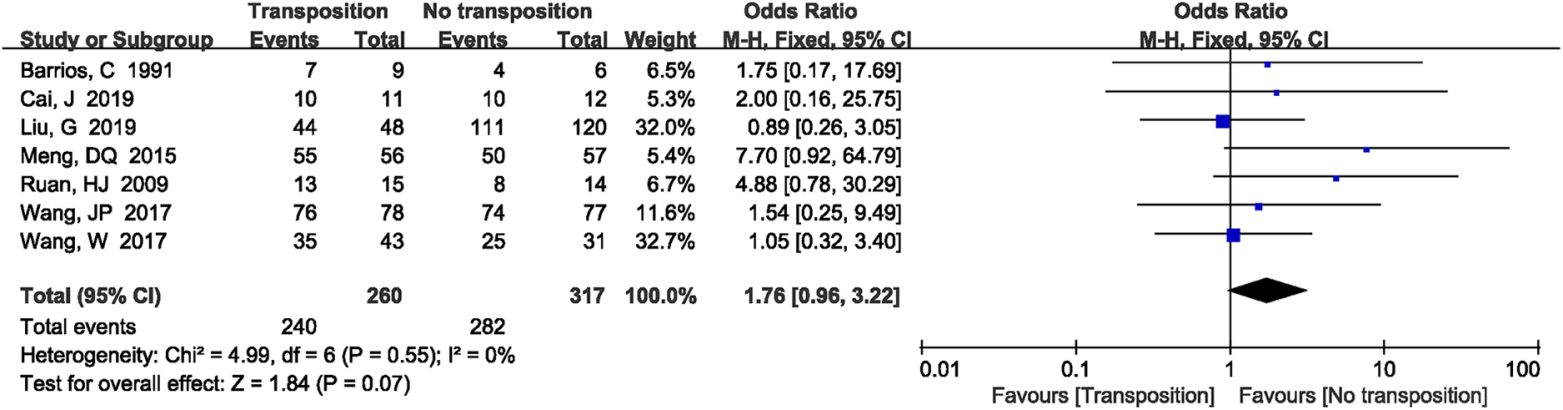
A forest plot showing the elbow joint function.

A total of 7 studies (9, 12–14, 19, 20, 22) reported elbow joint function. There was no significant heterogeneity in the study (*P *= 0.55, *I*^2^*^ ^*= 0%). Fixed effects model was performed. There was no significant difference between two groups (OR = 1.76, 95%CI: 0.96–3.22, *P *= 0.07, Figure 8).

### 3.5. Safety

#### 3.5.1. Ulnar neuritis

**Figure 9:**
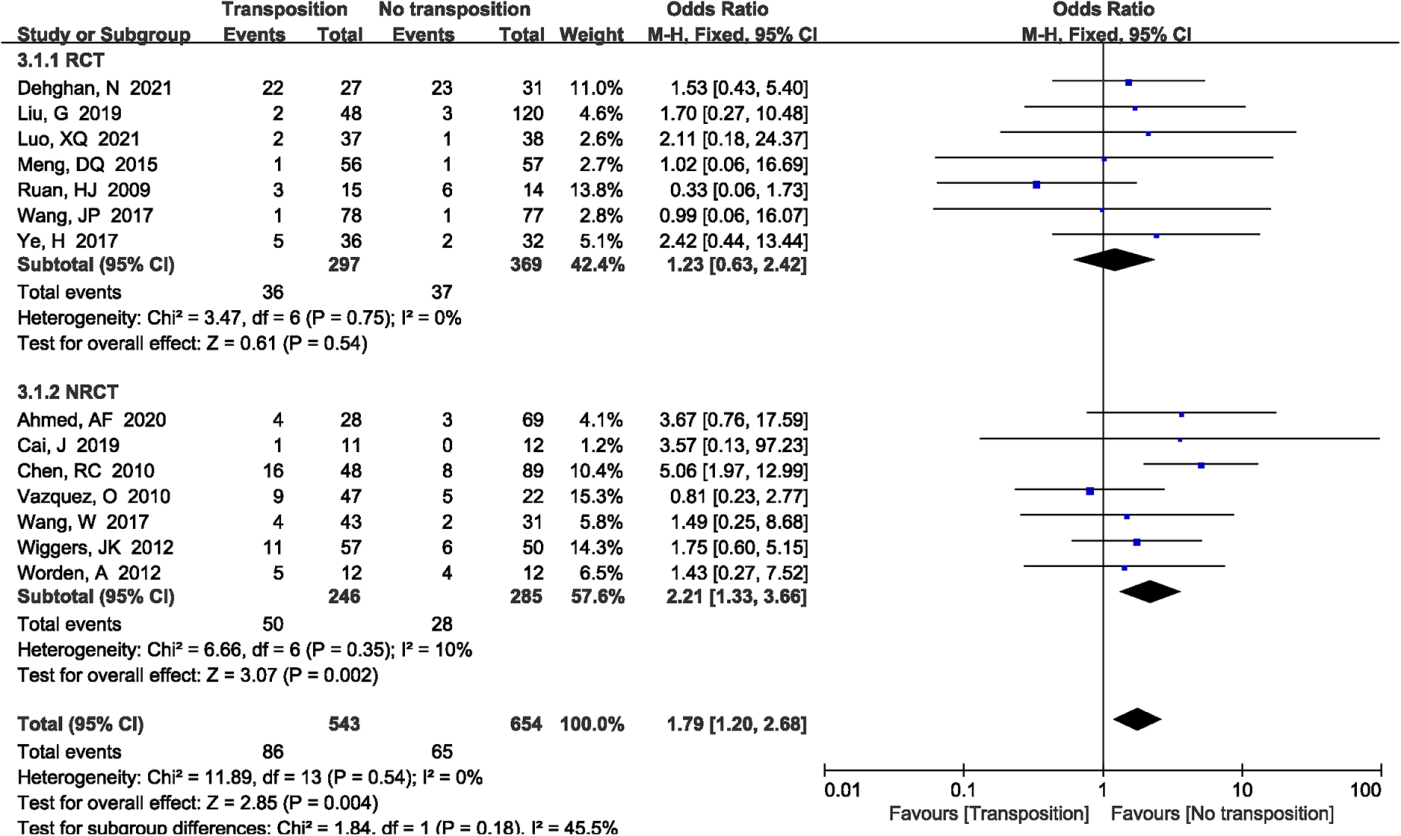
A forest plot showing the ulnar neuritis.

A total of 14 studies (7–9, 11–15, 17, 19–21, 23, 24) reported the incidence of ulnar neuritis included in the study. The results demonstrated that non-transposition group had lower ulnar neuritis rate compared with anterior transposition group (OR = 1.79, 95%CI: 1.20–2.68, *P *= 0.004, Figure 9). There was no significant heterogeneity (*P *= 1.00, *I*^2^*^ ^*= 0%), and the fixed-effects model was performed. However, the subgroup analysis showed that there was no significant difference when only RCTs were included between two group (*P *= 0.54).

#### 3.5.2. Humerus nonunion

**Figure 10:**
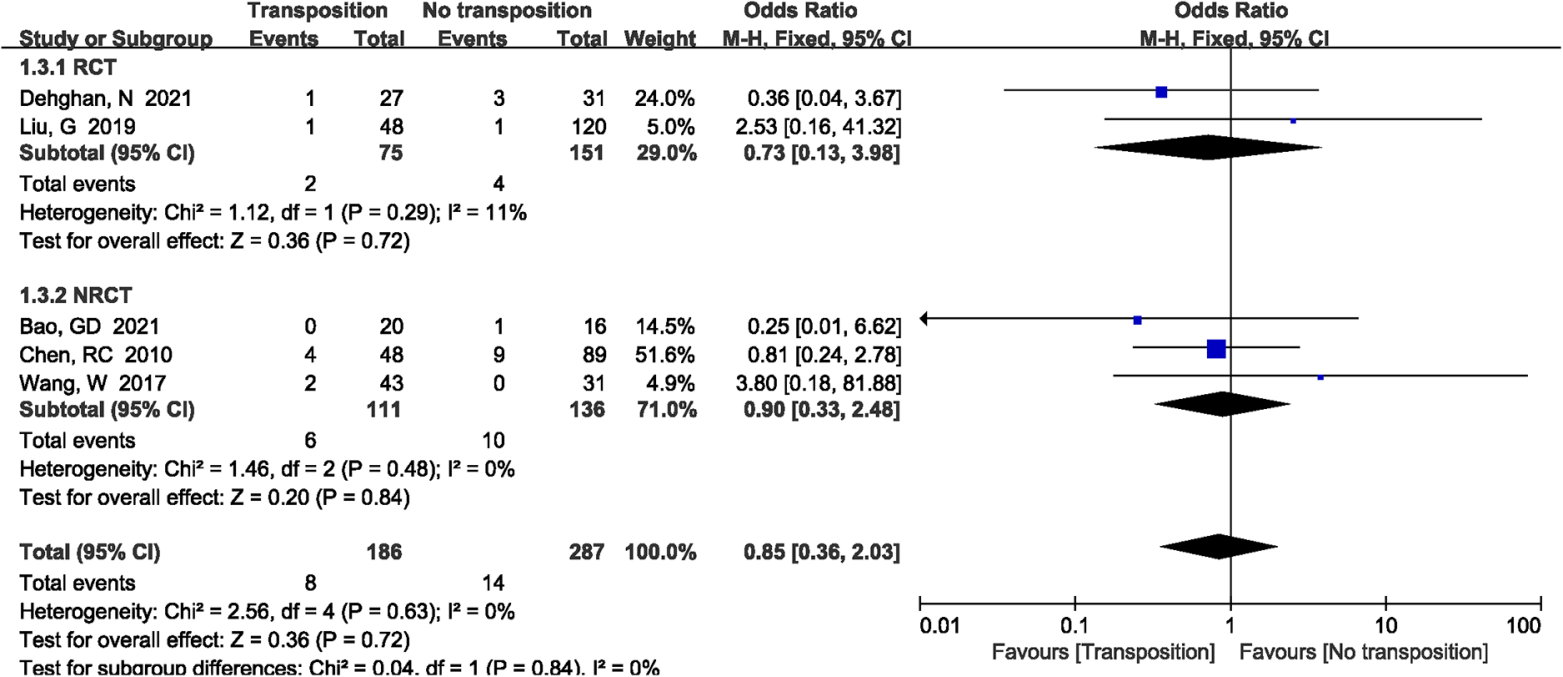
A forest plot showing the humerus nonunion.

A total of 5 studies (8, 14, 17, 18, 20) reported the incidence of humerus nonunion included in the study. The results demonstrated that there was no significant difference between two groups (OR = 0.85, 95%CI: 0.36–2.03, *P *= 0.72, Figure 10). There was no significant heterogeneity (*P *= 0.63, *I*^2^*^ ^*= 0%), and the fixed-effects model was performed.

#### 3.5.3. Infection

**Figure 11:**
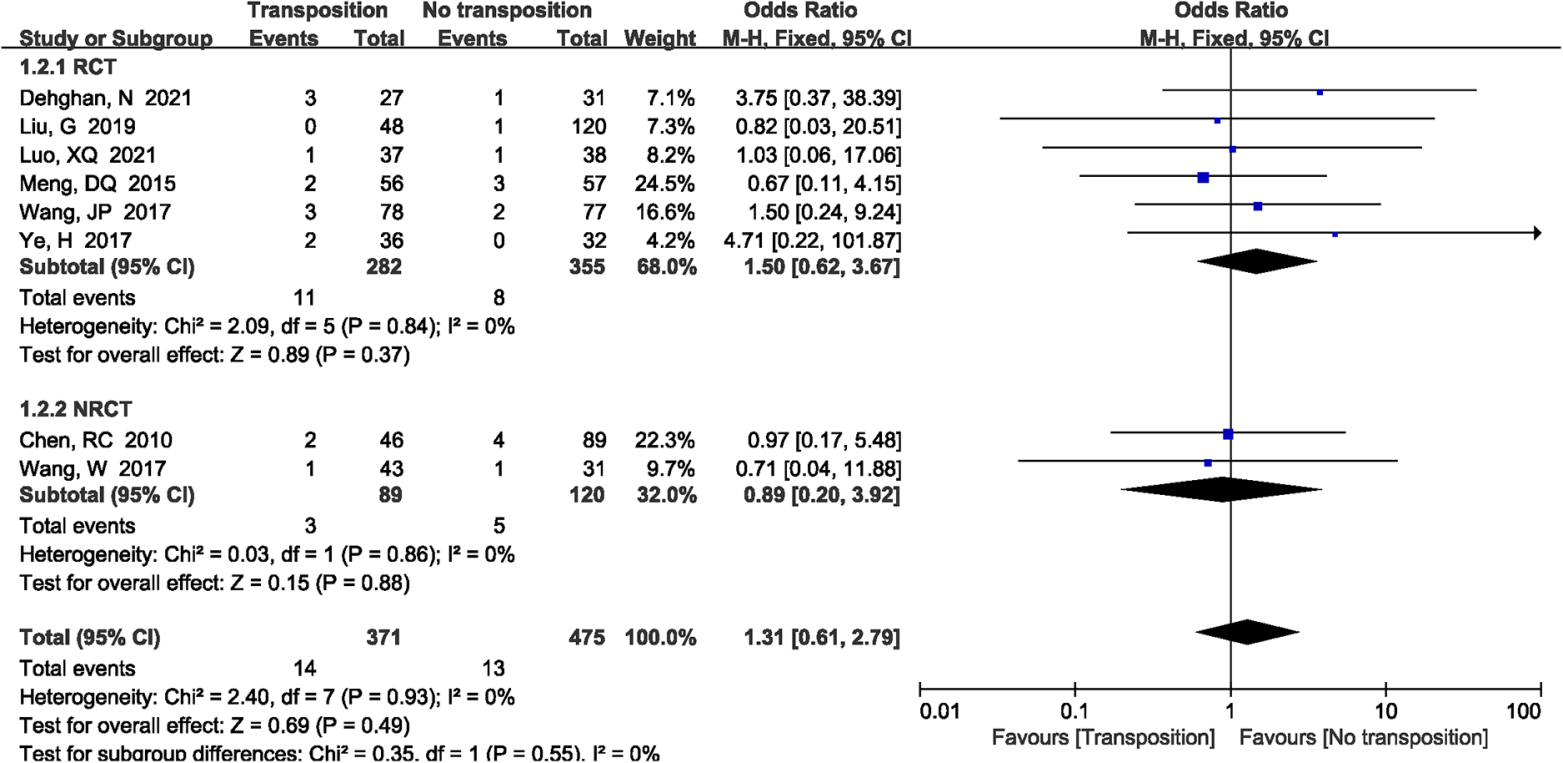
A forest plot showing the infection.

A total of 8 studies (8, 11–15, 17, 20) reported the incidence of infection included in the study. The results demonstrated that there was no significant difference between two groups (OR = 1.31, 95%CI: 0.61–2.79, *P *= 0.49, Figure 11). There was no significant heterogeneity (*P *= 0.93, *I*^2^*^ ^*= 0%), and the fixed-effects model was performed.

### 3.6. Publication bias

**Figure 12:**
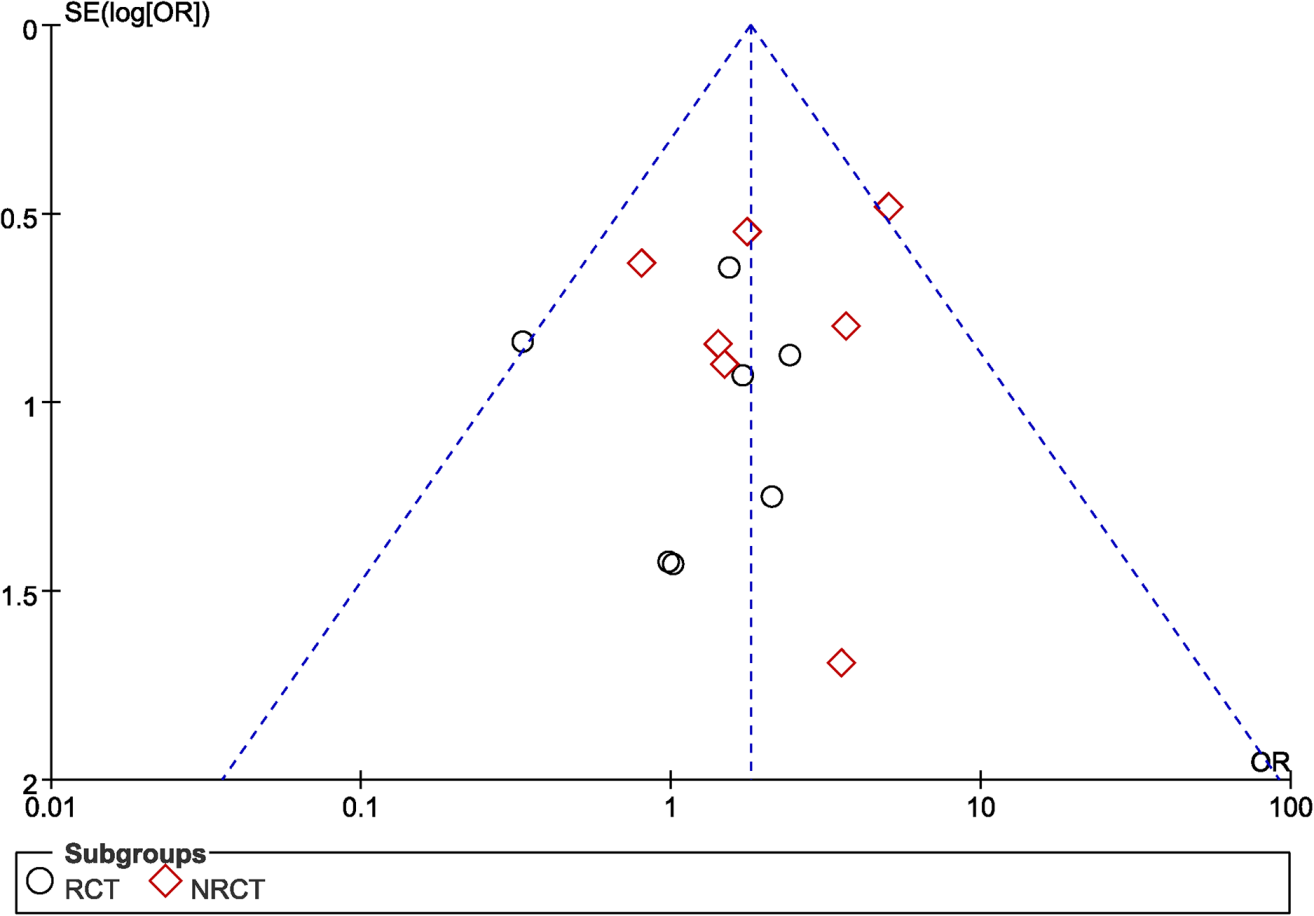
A funnel plot showing publication bias for ulnar neuritis.

The funnel plot was used to evaluate the publication bias of studies. For the study of ulnar neuritis rate (Figure 12), the funnel plot was not symmetrical. It indicated the possibility of publication bias.

## 4. Discussion

Despite advances in the management of distal humerus fractures, complications, such as ulnar neuropathy still pose a challenge to clinicians. Regardless of whether there was ulnar nerve injury before surgery, orthopedic surgeons should routinely dissociate the ulnar nerve before reducing the fracture to protect the ulnar nerve, fully expose the fracture site, and avoid the difficulties of internal fixation and implantation (25–27). At present, many scholars believed that if there is ulnar nerve injury before surgery, it is necessary to take an anterior transposition of the ulnar nerve to avoid compression of the ulnar nerve, which can reduce the occurrence of postoperative ulnar neuritis (28, 29). However, in recent years, there were many different opinions on whether anterior transposition of the ulnar nerve was necessary in patients without ulnar nerve injury before surgery, as some study reported higher incidence of ulnar neuritis when transposition was performed (8). According to these scholars, compared with the anterior transposition of the ulnar nerve, only in site placement of the ulnar nerve could not only simplify surgery procedures, but also reduce the probability of iatrogenic injury. Therefore, the intraoperative management of the ulnar nerve in patients without preoperative ulnar nerve injury was controversial. The purpose of this meta-analysis was to evaluate the benefits and risks by comparing anterior transposition of the ulnar nerve with non-transposition treatment for patients with distal humerus fractures.

Our meta-analysis demonstrated that transposition group and non-transposition group had no statistical significance in blood loss, hospital stays, and fracture healing time (respectively *P *= 0.31, *P *= 0.11 and *P *= 0.33). However, we found that non-transposition group had shorter operation time than transposition group (*P* < 0.00001). Compared with *in situ* placement of the ulnar nerve, anterior transposition of the ulnar nerve was an additional surgical procedure on the basis of ORIF, so the operation time was relatively longer. Anterior transposition of ulnar nerve did not increase blood loss, hospital stays, and fracture healing time, as these were often related to the degree of fracture, soft tissue injury, and the level of operation of the surgeon (30). What's more, we found that there was no heterogeneity after subgroup analysis for operation time. However, blood loss, hospital stays, and fracture healing time had a large heterogeneity, and subgroup analysis also found no the source of heterogeneity. Therefore, the source of heterogeneity is mainly related to the difference of surgical level among different surgeons.

After distal humerus injury, there was a large number of local bone defect, and there was a risk of delayed or even non-union (31, 32). Some studies have shown that the delay fracture recovery rate could reach 2%–10% after ORIF (33). What's more, Chen RC et al. (8) reported that anterior transposition group and non-transposition group had no statistical difference in nonunion (respectively, 9.1% vs. 11.3%, *P *= 0.73). Our meta-analysis also demonstrated that anterior transposition group was not superior to non-transposition group in fracture nonunion. Fracture nonunion often has a certain relationship with infection. Due to the severe soft tissue injury of the elbow joint, the relatively thin soft tissue envelope, and the shear forces generated during early exercise, there is a risk of serious wound complications in the elbow joint after surgery (34). Lawrence et al. reported 89 distal humerus fractures which were treated with internal fixation. Fourteen patients (15.7%) developed a severe wound complication requiring on average 2.5 (rang, 1–6) additional surgical procedures (6). A multiple-center randomized controlled trial also demonstrated that 3 patients in the transposition group and 1 patients in the situ group had superficial wound infections (respectively, 11.11% vs. 3.23%), which was no statistical difference between two groups (17). Our meta-analysis also found same clinical outcomes for wound infections between two groups.

Three literatures evaluated elbow joint function according to Mayo scores, and four literatures evaluated elbow joint function according to Cassebaum score. The Mayo Elbow Performance Score (MEPS) and Cassebaum score were used as an objective measure of overall outcome by assessing motion ability, joint stability and pain level (35, 36). Most of the studies on improving postoperative elbow function focus on surgical approach, internal fixation device and functional rehabilitation exercise. Some studies had reported that the average MEPS score was slightly better in the triceps sparing approach group (86.56 ± 10.66) compared with olecranon osteotomy approach group (83.57 ± 10.96) but it was statistically not significant (*P* = 0.289) (30). And postoperative rehabilitation exercise was an important treatment that could not be ignored. Early functional exercise could prevent joint ossification and bone loss, and could accelerate the recovery of elbow function (37). Therefore, our meta-analysis also found that anterior transposition group was no superior non-transposition group in elbow joint function and elbow joint motion (respectively, *P *= 0.07 and *P *= 0.59).

Treatment of the ulnar nerve remains an unsettled issue. Some studies showed a complication rate of up to 44% in patients with distal humerus fracture after internal fixation, including neuropathies, mechanical failure and wound dehiscence (38). However, the ulnar neuropathy as a complication of distal humerus fracture has been reported with a magnitude ranging from 0% to 51% (7, 39). Shin et al. reported a 22% rate of postoperative ulnar nerve palsies despite performing adequate release and nerve transposition (40). The ulnar neuropathies were caused by the scar tissue development, thickening in the fibro-osseous tunnel, and swelling. Although anterior transposition nerve could reduce the incidence of postoperative ulnar neuritis, some authors found that anterior transposition ulnar nerve actually increased the incidence of postoperative ulnar neuritis. Because anterior transposition of the ulnar nerve had longer operation time compared with *in situ* placement group, which might increase the duration of the tourniquet. Thus, the ulnar nerve ischemia and hypoxia time are prolonged, which increased the incidence of ulnar neuritis. In a recent meta-analysis, it found that incidence of neuropathy in the transposition group was higher (23.5%) as compared with the *in situ* group (15.3%) as it only included five retrospective studies (41). What's more, our meta-analysis also showed that anterior transposition group had higher the incidence of postoperative ulnar neuritis than non-transposition group (*P *= 0.004). However, the subgroup analysis showed that there was no significant difference when only RCTs were included between two group (*P* = 0.54). What's more, randomized controlled trials have a higher level of experimental evidence, and it can reduce selection bias, report bias, and observation bias. Therefore, the meta-analysis demonstrated that non-transposition group did not add the incidence of postoperative ulnar neuritis compared with anterior transposition group.

However, there are also a few limitations in our study: (1) Some pooled results from included studies were strongly subjective, which may influence the results due to the different experiences from doctors. (2) Most of the included studies were retrospective studies, which have a great impact on the experimental results. (3) Inclusion and exclusion criteria of some studies are different. (4) Heterogeneity among the included studies was unavoidable because of racial differences, age difference, and fracture type, and surgical approach. Therefore, physicians around the world should be careful to interpret our results in clinical practice.

## 5. Conclusion

Current evidence suggests that anterior transposition of the ulnar nerve is not superior to non-transposition of the ulnar nerve for patients with distal humerus fractures. On the contrary, non-transposition group had shorter operation time than anterior transposition group and non-transposition group might decrease the incidence of postoperative ulnar neuritis. In patients with postoperative ulnar neuropathy after ORIF of the distal humerus, anterior transposition and non-transposition groups seems to be an effective treatment. Considering that dynamic stabilization device group also has its limitations, large sample, double-blind and multi-center RCTs are needed to verify our conclusion.

## Data Availability

The original contributions presented in the study are included in the article/Supplementary Material, further inquiries can be directed to the corresponding author/s.
